# Renieramycin T Induces Lung Cancer Cell Apoptosis by Targeting Mcl-1 Degradation: A New Insight in the Mechanism of Action

**DOI:** 10.3390/md17050301

**Published:** 2019-05-21

**Authors:** Korrakod Petsri, Supakarn Chamni, Khanit Suwanborirux, Naoki Saito, Pithi Chanvorachote

**Affiliations:** 1 Cell-Based Drug and Health Products Development Research Unit, Chulalongkorn University, Bangkok 10330, Thailand; korrakod.petsri@gmail.com; 2 Doctor of Philosophy Program in Interdisciplinary Pharmacology, Graduate School, Chulalongkorn University, Bangkok 10330, Thailand; 3 Department of Pharmacology and Physiology, Faculty of Pharmaceutical Sciences, Chulalongkorn University, Bangkok 10330, Thailand; 4 Department of Pharmacognosy and Pharmaceutical Botany, Faculty of Pharmaceutical Sciences, Chulalongkorn University, Bangkok 10330, Thailand; supakarn.c@pharm.chula.ac.th (S.C.); khanit.s@pharm.chula.ac.th (K.S.); 5 Graduate School of Pharmaceutical Sciences, Meiji Pharmaceutical University, 2-522-1 Noshio, Kiyose, Tokyo 204-8588, Japan; naoki@my-pharm.ac.jp

**Keywords:** Renieramycin T, *Xestospongia* sp., Lung cancer, Anti-cancer, Marine sponge, Mcl-1 degradation

## Abstract

Among malignancies, lung cancer is the major cause of cancer death. Despite the advance in lung cancer therapy, the five-year survival rate is extremely restricted due to therapeutic failure and disease relapse. Targeted therapies selectively inhibiting certain molecules in cancer cells have been accepted as promising ways to control cancer. In lung cancer, evidence has suggested that the myeloid cell leukemia 1 (Mcl-1) protein, an anti-apoptotic member of the Bcl-2 family, is a target for drug action. Herein, we report the Mcl-1 targeting activity of renieramycin T (RT), a marine-derived tetrahydroisoquinoline alkaloid that was isolated from the Thai blue sponge *Xestospongia* sp. RT was shown to be dominantly toxic to lung cancer cells compared to the normal cells in the lung. The cytotoxicity of this compound toward lung cancer cells was mainly exerted through apoptosis induction. For the mechanism of action, we found that RT mediated activation of p53 protein and caspase-9 and -3 activations. While others Bcl-2 family proteins (Bcl-2, Bak, and Bax) were minimally changed in response to RT, Mcl-1 protein was dramatically diminished. We further performed the cycloheximide experiment and found that the half-life of Mcl-1 was significantly shortened by RT treatment. When MG132, a potent selective proteasome inhibitor, was utilized, it could restore the Mcl-1 level. Furthermore, immunoprecipitation analysis revealed that RT significantly increased the formation of Mcl-1-ubiquitin complex compared to the non-treated control. In conclusion, we report the potential apoptosis induction of RT with a mechanism of action involving the targeting of Mcl-1 for ubiquitin-proteasomal degradation. As Mcl-1 is critical for cancer cell survival and chemotherapeutic failure, this novel information regarding the Mcl-1-targeted compound would be beneficial for the development of efficient anti-cancer strategies or targeted therapies.

## 1. Introduction

Lung cancer causes nearly 30% of all cancer deaths globally. Despite the advance in lung cancer therapy, most patients hardly survive longer than five years after the first time diagnosis due to the high drug resistance and metastasis [1]. In recent years, targeted therapies aiming to selectively inhibit certain receptors or proteins influencing growth and survival of cancer cells have been recognized as highly promising treatments to control cancer [2].

B-cell lymphoma 2 (Bcl-2) family proteins are among the most important protein groups that dominate the apoptosis of cells. A number of studies have specified Bcl-2 family proteins as the crucial targets of anti-cancer drugs as well as gene therapy [3,4]. Besides, anti-apoptotic members of the Bcl-2 family (i.e., Bcl-2 and Mcl-1) are demonstrated to be involved in chemotherapeutic resistance [5,6,7]. Recent evidence has suggested that the survival of human cancers is likely to be dependent on expression levels and function of the myeloid cell leukemia 1 (Mcl-1) protein [8,9]. Mcl-1 is a member of the Bcl-2 family proteins with a prominent activity in apoptosis inhibition. The pro-survival function of Mcl-1 is due to the binding activity of the protein to pro-apoptotic members of the Bcl-2 family proteins, thus suppressing the activation of the apoptosis cascade [10,11,12,13]. In several cancers, Mcl-1 was frequently found amplified or overexpressed and, in particular, the augmented expression of Mcl-1 reflected the poor prognosis of many malignancies including lung cancer [14,15,16]. Since Mcl-1 is potentially the main contributor to multidrug resistance, this protein is highlighted as a principal target of drug action in the treatment of lung cancer.

In lung cancer, Mcl-1 has been shown to be a promising target of drug action [14,16]. Not only is its increased expression critical for oncogenesis and cancer progression, but Mcl-1 is also involved in conferring chemotherapeutic drug resistance in this cancer [17,18,19]. Mcl-1 is a relatively unstable protein, and the degradation of Mcl-1 can be induced by certain anti-cancer drugs [20,21,22,23]. Intracellular Mcl-1 level is tightly regulated by the ubiquitin-proteasomal degradation mechanisms. Therefore, compounds with potent activity in eliminating Mcl-1 in cancer cells are of interest as good candidates for Mcl-1-targeted therapy. 

**Figure 1:**
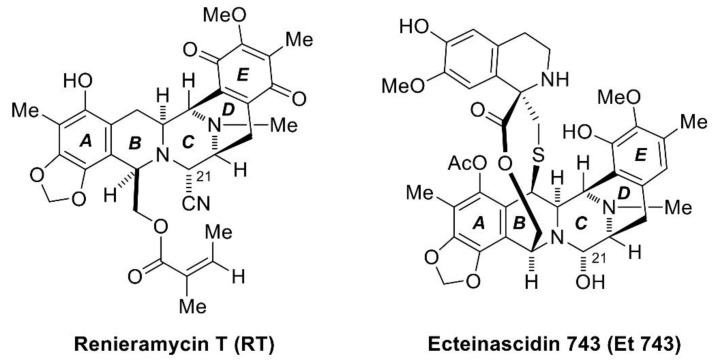
The structures of renieramycin T (RT) and ecteinascidin 743 (Et 743).

The marine environment represents a countless and diverse resource for many potent bioactive compounds, which have recently been used for new drug developments to treat major diseases such as infection and cancer. Recently, antimicrobial, antitumor, and anti-inflammatory effects have been reported. The number of scientific publications on marine compounds has followed an upward trend in the last twenty years, especially in the field of cancer [24]. From many studies, the marine environment has produced a large number of very potent agents, which are able to inhibit the growth of human cancer cells and exhibit anticancer activities [25]. It has been found that substances from marine organisms have structural and chemical features generally not found in terrestrial natural products; their structures have more complexity and diversity [26,27]. Thus, these marine-derived molecules are capable of interacting with numerous biomolecular targets to either inhibit or promote specific biological functions against various types of cancer cell lines. One of the marine-derived natural products is renieramycins. Renieramycins are alkaloids in the tetrahydroisoquinoline family [28], which is derived from various marine organisms, including sponges in the genera *Reniera* [29,30], *Xestospongia* [31,32,33,34,35], *Cribrochalina* [36,37], and *Neopetrosia* [38]. However, they are unstable and decomposed after extraction and isolation. Therefore, a very unstable amino alcohol functionality at C-21 in their structure is converted into stable aminonitrile compounds by pretreatment with potassium cyanide [30,39]. In this work, we have focused on renieramycin T (RT), which is the renieramycin-related compound isolated from the Thai blue sponge *Xestospongia* sp. Normally, the A and E rings in almost all renieramycin structures are in the quinone form, but RT has a substituted aromatic A-ring having a dioxymethylene moiety [39], which is the same as ecteinascidins, another tetrahydroisoquinoline compound isolated from marine tunicates [28,40]. Thus, RT is the renieramycin–ecteinascidin hybrid in the tetrahydroisoquinoline alkaloid family (Figure 1).

## 2. Results

### 2.1. The Cytotoxicity and Apoptosis-Inducing Effect of Renieramycin T

**Figure 2:**
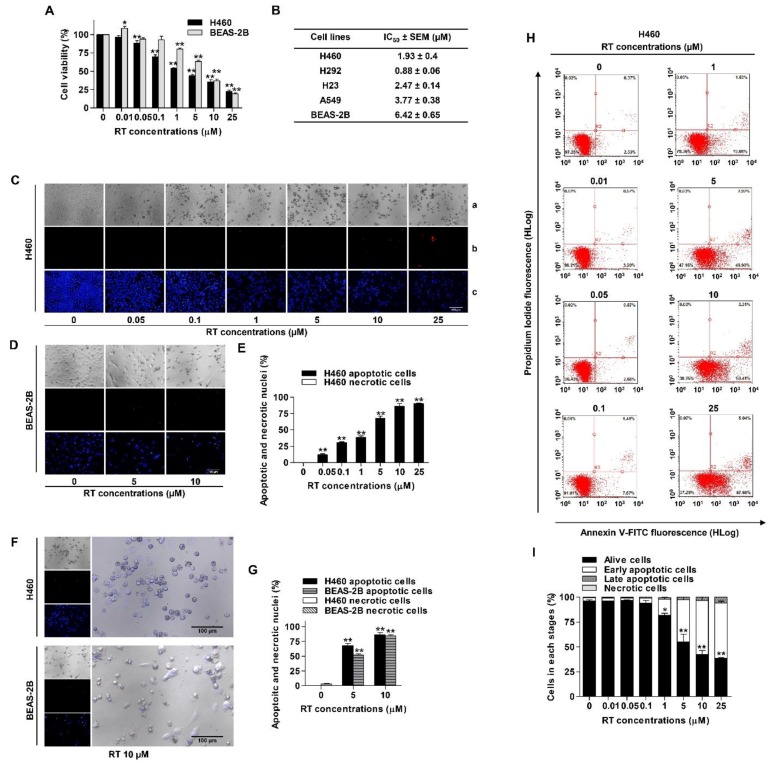
Renieramycin T (RT) reduced cell viability and induced apoptosis in NSCLC and human normal lung epithelial (BEAS-2B) cell lines. (**A**) NSCLC and BEAS-2B cell lines were treated with various concentrations of RT (0–25 µM) for 24 h. Percentages of cell viability were determined using the MTT assay. (**B**) The half maximal inhibitory concentrations (IC_50_) in NSCLC and BEAS-2B cell lines were calculated by comparison with the untreated control. (**C**–**G**) H460 and BEAS-2B cell lines were treated with RT (0–25 µM) for 24 h. Hoechst 33342 and propidium iodide (PI) were added. Then, Images were detected by using an inverted fluorescence microscope (a–c). A condensed blue fluorescence of Hoechst 33342 reflected fragmented chromatin in apoptotic cells (c) while a red fluorescence of PI reflected late apoptotic or necrotic cells (b) comparing with no staining condition (a). Percentages of nuclear fragmented and PI positive cells were calculated. (**H**) H460 was treated with RT (0–25 µM) for 24 h. Apoptotic and necrotic cells were determined using annexin V-FITC/PI staining with flow cytometry. (**I**) Percentages of cells at each stage were calculated. Data represented the mean ± SEM (n = 3) (* 0.01 ≤ *p* < 0.05, ** *p* < 0.01, compared with the untreated control).

To elucidate the anti-cancer potential of RT, we first determined the cytotoxic profile of RT in several non-small cell lung cancer (NSCLC) cells including H460, H292, H23, and A549 cell lines and human bronchial epithelial cells (BEAS-2B) were used for comparison. Cell viability was evaluated by the 3-(4, 5-dimethylthiazolyl-2)-2, 5-diphenyltetrazolium bromide (MTT) assay. Cells were incubated with various concentrations of RT (0–25 µM) for 24 h. The results showed that RT significantly reduced cell viability in both NSCLC and human bronchial epithelial cell lines in a concentration-dependent manner compared with untreated controls (Figure 2A,B). The cytotoxic effects of RT were found to be significant at the concentration of 0.05 µM in all NSCLC cells, while the cytotoxicity of the compound on BEAS-2B was found at 1 µM. IC_50_ values of RT were determined in various lung cancer cells, and the results are presented in Figure 2B, which indicate that the IC_50_ of RT in NSCLC cells was generally lower than that of normal epithelial cells. Overall, these results suggest the promising effect of RT as an alternative approach in anti-cancer therapy.

The mode of cell death was further evaluated by monitoring the morphology of the cell nucleus as well as cell membrane integrity. Hoechst 33342 staining was used to evaluate the nucleus morphology of apoptotic cells, while the propidium iodide (PI) stained the cell’s necrotic features. H460 and BEAS-2B were treated with RT at various concentrations (0–25 µM) for 24 h, and the cells were co-stained with Hoechst 33342 and PI. Condensed or fragmented nuclei exhibiting blue fluorescence of Hoechst 33342 represented fragmented chromatin in apoptotic cells while a red fluorescence of PI indicated necrotic cells. The results revealed that RT caused an increase in apoptosis in a dose-dependent manner, whereas necrotic cells exhibited minimal in response to all treatments (Figure 2C–E). Figure 2F shows the morphology of the cells in H460 and BEAS-2B cells treated with 10 µM RT. We observed that RT could kill lung cancer cells and resulted in the rounded apoptosis cells; however, the normal BEAS-2B showed a relatively higher amount of living intact cells (Figure 2G).

To confirm, flow cytometric analysis of apoptosis and necrosis using annexin V-FITC/PI staining was utilized. H460 was similarly treated with RT (0–25 µM) for 24 h. The results showed that RT dramatically induced apoptosis in a concentration-dependent manner (Figure 2H,I). As shown in Figure 2H, percentages of early apoptotic cells in response to 1, 5, 10, and 25 µM of RT were 19.08%, 49.90%, 58.41%, and 57.60%, respectively. 

### 2.2. Renieramycin T Promotes Apoptosis by Targeting Mcl-1 to Proteasomal Degradation

**Figure 3:**
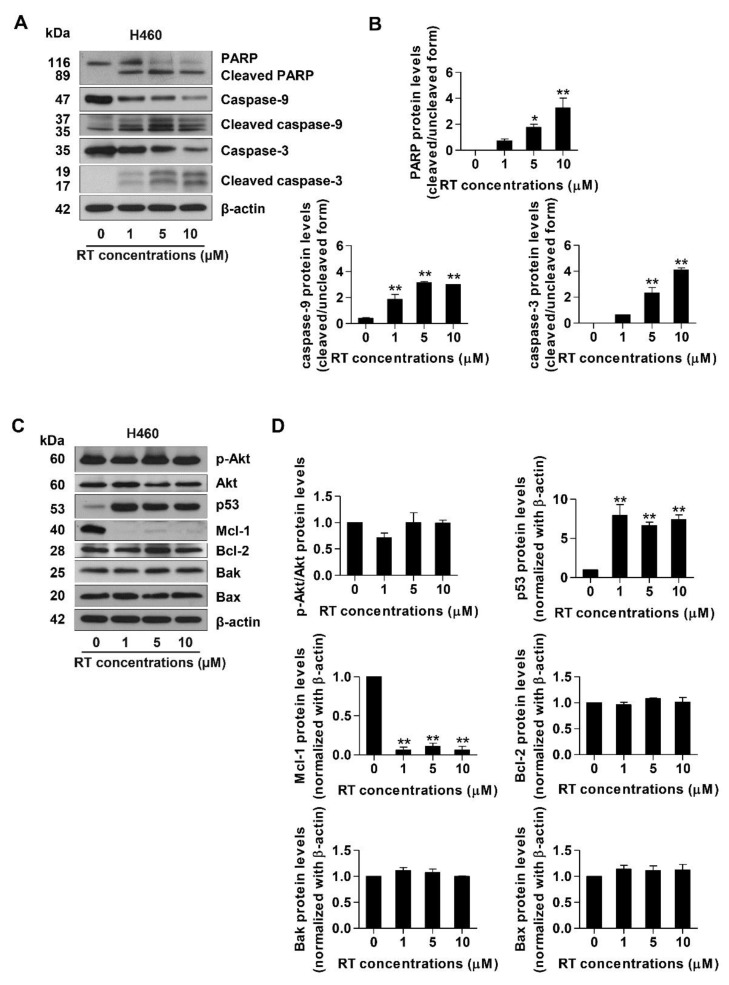
Renieramycin T (RT) induced activation of PARP, caspase-3, and caspase-9 in the H460 cell line. Moreover, RT also significantly up-regulated p53 and down-regulated Mcl-1 in the H460 cell line. H460 cells were treated with RT (0–25 µM) for 24 h. (**A**) and (**C**) Apoptotis-related proteins were measured with Western blot analysis. The blots were reprobed with β-actin to confirm equal loading of each of the protein samples. (**B**) and (**D**) The relative protein levels were calculated by densitometry. Data represented the mean ± SEM (n = 3) (* 0.01 ≤ *p* < 0.05, ** *p* < 0.01, compared with the untreated control).

In order to further investigate the mechanism of action of RT, specific apoptotic markers including caspase-3, caspase-9, Poly (ADP-ribose) polymerase (PARP), and their cleaved forms, as well as the up-stream apoptosis regulatory proteins were investigated by Western blot analysis. Lung cancer cells were treated with RT of 1–10 µM concentrations or left untreated for 24 h. Western blotting revealed that in response to RT treatment, the activated caspase-3 and caspase-9 significantly increased compared with the untreated control. Likewise, the cleaved form of PARP was up-regulated simultaneously (Figure 3A,B). 

For apoptosis induction, the key pro-survival protein Akt, its active form, phosphorylated Akt (pAkt), p53, and members of the Bcl-2 family proteins were monitored in RT-treated H460 cells. We found that treatment of the cells with RT did not alter the level of Akt and pAkt proteins. The underlying mechanism of apoptosis induction was further evaluated by investigating major regulators of p53-dependent apoptosis such as p53, anti-apoptotic proteins (Mcl-1 and Bcl-2), and pro-apoptotic proteins (Bak and Bax). The H460 cell line was treated in the same condition as the previous experiments. The results from Western blot analysis revealed that RT dramatically up-regulated p53. Interestingly, while the downstream targets of p53, including anti-apoptotic Bcl-2 and pro-apoptotic Bax and Bak, were found to be only slightly altered in response to RT treatment, the anti-apoptotic Mcl-1 was found to be dramatically depleted (Figure 3C,D). As Mcl-1 is known to be critical for cancer cell survival [8,9], this result implies that the principal mechanism of action of RT may involve the targeting of the Mcl-1 function.

### 2.3. Renieramycin T Decreases Mcl-1 Through the Induction of Mcl-1 Proteasomal Degradation

Mcl-1 was not only shown to protect cells against apoptotic stimuli, but also this protein has been suggested as an important therapeutic target in human cancers [41]. As targeting Mcl-1 for cell degradation has been demonstrated as a promising action of novel anti-cancer drugs [15,42,43], we further evaluated the effect of RT on Mcl-1 stability in lung cancer cells. To compare the Mcl-1 stability between RT-treated and control cells, the cycloheximide (CHX) chasing assay was used followed by Western blot analysis. CHX, an inhibitor of protein biosynthesis, was widely used for determining the half-life of the protein of interest [44]. Therefore, H460 cells were treated with RT (0–5 µM) in the presence or absence of 50 µg/mL CHX and the level of Mcl-1 over time was determined. 

**Figure 4:**
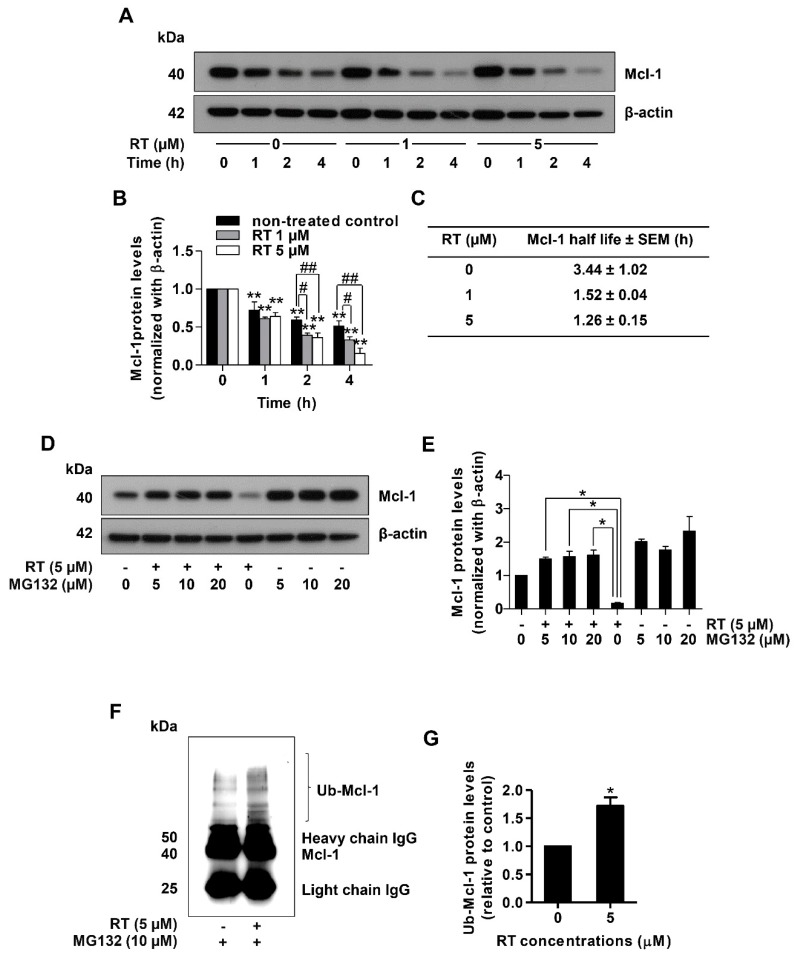
Renieramycin T (RT) induced ubiquitin-mediated Mcl-1 proteasomal degradation. (**A**) Cycloheximide (CHX) chasing assay was performed to measure Mcl-1 half-lives. H460 cells were treated with RT (0–5 µM) with or without 50 µg/mL CHX as indicated by the time in h. Western blot analysis was performed for determined Mcl-1 levels. The blots were reprobed with β-actin to confirm equal loading of each of the protein samples. (**B**) The relative protein levels were calculated by densitometry (** *p* < 0.01, compared with the untreated control at 0 h, # 0.01 ≤ *p* < 0.05, ## *p* < 0.01, compared with the untreated control at the same time). (**C**) Mcl-1 half-lives were calculated. (**D**) H460 cell line was treated with RT (0–5 µM) with or without MG132 (0–20 µM) for 4 h. Mcl-1 expression levels were measured using Western blot analysis. The blots were reprobed with β-actin to confirm equal loading of each of the protein samples. (**E**) The reversal of RT-mediated down-regulation of Mcl-1 levels by MG132 was calculated by densitometry compared to the non-MG132 treated group (* 0.01 ≤ *p* < 0.05, compared with the non-MG132 treated group). (**F**) H460 was treated with RT (5 µM) and MG132 (10 µM) for 4 h. Then, protein lysates were collected subsequent to Mcl-1 immunoprecipitation, and the ubiquitinated protein levels were measured by Western blotting. (**G**) Ub-Mcl-1 levels were quantified using densitometry (* *p* < 0.01, compared with the untreated control) All data represented the mean ± SEM (n = 3).

Figure 4A,B shows that in the condition where protein production was blocked, RT decreased the stability of the Mcl-1 protein. The difference was first detected at 4 h after RT treatment (Figure 4A,B). We also determined the Mcl-1 protein half-life and found that the half-life of Mcl-1 protein in the RT-treated groups was about 1.4 h, whereas in the untreated control the value was 3.4 h (Figure 4C). To prove that this decrease in Mcl-1 stability was through proteasomal degradation of the protein, we utilized MG132, a potent selective proteasome inhibitor. Treatment of the lung cancer cells with RT for 4 h dramatically decreased Mcl-1 level, while the addition of MG132 (0–20 µM) could restore the Mcl-1 level (Figure 4D,E). Taken together, it could be concluded that RT induced Mcl-1 proteasomal degradation. Importantly, it was shown that the degradation of Mcl-1 protein required ubiquitination [45]. The tagging of ubiquitin and poly-ubiquitin molecules on the target proteins conferred the recognition of the proteasome providing a specific degradation machinery of certain functional proteins. We also provided the evidence of Mcl-1 ubiquitination using immunoprecipitation and analysis of the Mcl-1-ubiquitin complex (Ub-Mcl-1) in the lung cancer cells treated with RT and non-treated control cells. After treating the cells with 5 µM RT for 4 h, the Mcl-1 protein was selectively isolated from cell lysates by the Mcl-1 antibody. Then, unwanted proteins were washed out and only Mcl-1 complex was analyzed for ubiquitin through Western blot analysis. Figure 4F,G shows that treatment of the cells with RT dramatically enhanced the formation of the Mcl-1-ubiquitin complex compared to the baseline complex in non-treated controls. Taken together, the mechanism of action of RT is through the induction of Mcl-1 degradation via ubiquitin-proteasomal degradation. 

## 3. Discussion

Apoptosis is a critical component in cancer pathogenesis. The origin of cancer, as well as its progression, depends on the dysregulation or disruption of proliferation and apoptosis. The tumor suppressor p53 has a principal role in the control of DNA repair, cell cycle arrest, and apoptosis [46,47]. In response to DNA-damage, p53 is activated and most probably exerts its transcriptional regulatory functions to control the level of Bcl-2 family proteins, causing the increased cellular level of pro-apoptotic members of the Bcl-2 family and in a concomitant decrease of the anti-apoptotic proteins [48]. Then, the death-survival threshold is altered by competitive dimerization between anti- and pro-apoptotic proteins. The pro-apoptotic dimers could create the release of mitochondrial contents to the cytoplasm, and such contents activate the function of caspases leading to apoptosis [49]. We found that the treatment of the lung cancer cells with RT resulted in the significant induction of p53 that may, at least in part, play a role in RT-mediated apoptosis (Figure 3C,D). Interestingly, our protein analysis shows a striking effect on the cellular protein level of Mcl-1 (Figure 3C,D). Mcl-1 is an anti-apoptotic protein that has garnered increased attention in lung cancer cell biology as it was shown to be highly expressed in lung cancer [14,16]. Besides, Mcl-1 was shown to be important for the survival of lung cancer cells [50] and the multivariate analysis indicated MCl-1 expression to be a significant prognostic factor of lung cancer [14,51]. 

RT is a member of tetrahydroisoquinoline marine alkaloids in the renieramycins series from the *Xestospongia* sp. found in Thailand and the Philippines [39]. Evidence has shown that renieramycins have strong cytotoxicity against various cancer cell lines, including breast (T47D), colon (HCT116) and DLD1), lung (QG56 and H460), pancreatic (AsPC1), and prostate (DU145) human carcinomas [31,34,39,52]. Interestingly, RT, the renieramycin–ecteinascidin hybrids in the tetrahydroisoquinoline alkaloid family, showed strong cytotoxicity in several cancer cell lines including colon (HCT116), prostate (DU145), non-small cell lung (H292, H460 and QG56), breast (T47D), and pancreatic (AsPC1) cancers [39,53,54,55]. Moreover, our previous study suggested that 5-*O*-acetyl-renieramycin T, a modified compound of RT, exerted a potential to suppress cancer stem cell (CSCs) growth, which is represented by a decrease in the CSCs markers CD44 and CD133 due to depletion of the protein kinase B (AKT) signal resulting in apoptosis induction of CSCs [56]. However, cell death mechanisms in CSCs and cancer cells might be different since CSCs have special characteristics only found in the stem cell population such as metabolic activities, signaling pathways, and cell cycle regulation [57,58,59]. Although anti-CSC activities of 5-*O*-acetyl-renieramycin T have been reported, the mechanism of action of RT in lung cancer cells has not been elucidated. 

In an agreement with evidence favoring the use of Mcl-1 targeted therapy [15,41], we have shown herein that RT could induce Mcl-1 degradation in lung cancer cells. RT, an active compound from the blue sponge *Xestospongia* sp., was demonstrated here to possess a potent induction effect of Mcl-1-targeted degradation. The results revealed that while other Bcl-2 family proteins were not affected by the treatment with RT, the level of Mcl-1 was dramatically diminished (Figure 3C,D). In fact, the degradation process of Mcl-1 was shown to involve the ubiquitin-proteasomal pathway [45]. In addition, Mcl-1 degradation was shown to be important for targeted therapeutics [22]. In this study, we found that under RT treatment, the Mcl-1 half-life was dramatically shortened. The cycloheximide-based assay showed that the half-life of Mcl-1 in response to 1 µM RT was 1.52 h in comparison to 3.44 h in the non-treated control cells (Figure 4A,C). When the selective proteasome inhibitor (MG132) was applied, the Mcl-1 level in the RT-treated cells was significantly restored, indicating that the proteasomal degradation plays a key part in RT function (Figure 4E,F). Further, we monitored the levels of the Mcl-1-Ubiquitin complex and found that the formation of the complex dramatically increased in the RT-treated cancer cells (Figure 4G,H). Together, these results strongly support the conclusion that RT mediated cell death by targeting the degradation of Mcl-1. 

## 4. Materials and Methods

### 4.1. Reagents and Antibodies

Dulbecco’s Modified Eagle’s Medium (DMEM) medium, Roswell Park Memorial Institute (RPMI) 1640 medium, fetal bovine serum (FBS), penicillin/streptomycin, l-glutamine, phosphate-buffered saline (PBS), and trypsin-EDTA were obtained from Gibco (Grand Island, NY, USA). 3-(4,5-dimethylthiazol-2-yl)-2,5-diphenyltetrazoliumbromide (MTT), dimethyl sulfoxide (DMSO), Hoechst 33342, propidium iodide (PI), cycloheximide, MG132, and bovine serum albumin (BSA) were purchased from Sigma-Aldrich, Co. (St. Louis, MO, USA). The primary antibodies, PARP (#9532), caspase-9 (#9502), caspase-3 (#9662), p53 (#9282), Mcl-1 (#94296), Bcl-2 (#4223), Bak (#6947), Bax (#5023), Akt (#9272), phosphorylated Akt (#4060), and β-actin (#4970), were obtained from Cell Signaling Technology (Danvers, MA, USA). The primary antibody ubiquitin was obtained from Sigma-Aldrich, Co. (St. Louis, MO, USA). The respective secondary antibodies, anti-rabbit IgG (#7074), and anti-mouse (#7076), were obtained from Cell Signaling Technology (Danvers, MA, USA).

### 4.2. Isolation of Renieramycin T (RT)

Renieramycin T (RT) was isolated from its natural source, the Thai blue sponge *Xestospongia* sp. that was collected from Sichang Island, Chonburi Province, Thailand, in October 2016 with the assistance of the Aquatic Resources Research Institute, Chulalongkorn University. The characteristics of the fresh sponge, the light bluish-gray color, bulbous surface lobes, the numerous and moderate size of oscules, and the easily crumbled texture were identified [34]. The collected blue sponge was homogenized and suspended in the phosphate buffer solution (pH 7). After that, the suspension was pretreated with potassium cyanide (KCN) until the KCN concentration reached 10 mM. The suspension was stirred continuously for 6 h. Then, methanol was added and the mixture was macerated for 48 h. The methanol extract was filtered and concentrated under reduced pressure to acquire an aqueous methanolic solution. The processes of maceration with methanol and concentration were repeated three times. After that, the concentrated aqueous methanolic solutions were combined and extracted with ethyl acetate. The ethyl acetate fractions were combined and concentrated. Then, the crude extract was obtained as a dark brown gum. Purification of RT was performed by using silica gel chromatography with the gradient solvent mixture of hexane, ethyl acetate, and methanol. RT was obtained with 0.001% weight by dry weight of the blue sponge [34]. The resulting amorphous yellow powder showed the spectroscopic data of the RT that was matched with the previous report [39]. Renieramycin T was obtained as an orange amorphous solid. [α]${}_{D}^{25}$ +232.1 (*c* 0.0028, CHCl_3_); CD Δ ε (c: 17 mM, methanol, 20 °C) −1.2 (354), −1.2 (329), −1.1 (313), +0.9 (291), +3.5 (272), +1.2 (248), +0.1 (233); IR (KBr) 3449, 2953, 2924, 2853, 1717, 1653, 1616, 1460, 1375, 1307, 1234, 1151, 1093 cm^−1^; ^1^H-NMR (CDCl_3_, 300 MHz) δ 6.00 (1H, qq, *J* = 7.2, 1.5 Hz, 26-H), 5.92 (1H, d, *J* = 1.2 Hz, OCH_2_O), 5.85 (1H, d, *J* = 1.2 Hz, OCH_2_O), 4.42 (1H, dd, *J* = 11.4, 3.8 Hz, 22-Hb), 4.29 (1H, s, 5-OH), 4.16 (1H, t, *J* = 3.8 Hz, 1-H), 4.10 (1H, d, *J* = 2.4 Hz, 21-H), 4.00 (1H, overlapped, 22-Ha), 3.99 (1H, overlapped, 11-H), 3.99 (3H, s, 17-OCH_3_), 3.36 (1H, dd, *J* = 7.2, 2.4 Hz, 13-H), 3.24 (1H, ddd, *J* = 12.0, 2.7, 2.4 Hz, 3-H), 2.87 (1H, br d, *J* = 15.0 Hz, 4-Hα), 2.75 (1H, dd, *J* = 20.7, 7.2 Hz, 14-Hα), 2.29 (1H, d, *J* = 20.7 Hz, 14-Hβ), 2.29 (3H, s, NCH_3_), 2.11 (3H, s, 6-CH_3_), 1.94 (3H, s, 16-CH_3_), 1.85 (3H, dq, *J* = 7.2, 1.5 Hz, 27-H_3_), 1.69 (3H, br d, *J* = 1.5 Hz, 28-H_3_), 1.67 (1H, dd, *J* = 15.0, 12.0 Hz, 4-Hβ); ^13^C-NMR (CDCl_3_, 100 MHz) δ 186.1 (C-15), 182.8 (C-18), 167.1 (C-24), 155.4 (C-17), 144.9 (C-7), 144.7 (C-5), 141.7 (C-20), 139.8 (C-26), 136.9 (C-8), 135.8 (C-19), 129.0 (C-16), 126.8 (C-25), 117.5 (CN-21), 113.1 (C-10), 112.2 (C-9), 106.1 (C-6), 101.1 (OCH_2_O), 64.6 (C-22), 61.0 (17-OCH_3_), 59.7 (C-21), 56.4 (C-1), 56.3 (C-3), 55.0 (C-11), 54.8 (C-13), 41.5 (NCH_3_), 26.8 (C-4), 21.2 (C-14), 20.5 (C-28), 15.8 (C-27), 8.8 (6-CH_3_), 8.7 (16-CH_3_); FAB+ HRMS m/z 576.2344 ([M + H], calcd for C_31_H_34_N_3_O_8_, 576.2347).

### 4.3. Preparation of the RT Stock Solution

RT was prepared as a 50 mM stock solution by dissolving it in dimethyl sulfoxide (DMSO) solution and then stored at −20 °C. It was freshly diluted with medium to the desired concentrations before using. The final concentration of DMSO was less than 0.5% solution, which showed no signs of cytotoxicity.

### 4.4. Cell Lines and Culture

The human bronchial epithelial cell line (BEAS-2B) and human non-small cell lung cancer (NSCLC) cell lines (H460, H292, H23, and A549) were obtained from the American Type Culture Collection (Manassas, VA, USA). H460, H292, and H23 were cultured in Roswell Park Memorial Institute (RPMI) 1640 medium. BEAS-2B and A549 were cultured in DMEM medium. The medium was supplemented with 10% FBS, 2 mM L-glutamine, and 100 units/mL of each of penicillin and streptomycin at 37 °C with 5% CO_2_ in a humidified incubator. 

### 4.5. Cell Viability Aassay

NSCLC and BEAS-2B cells were seeded into 96-well plates (1 × 10^4^ cells/well) for overnight. Cells were treated with various concentrations of RT (0, 0.01, 0.05, 0.1, 1, 5, 10, and 25 µM) for 24 h. Then, 100 µL of MTT solution (400 µg/mL) was added in each well and incubated for 3 hours at 37 °C. Supernatants were replaced with 100 µL of DMSO to dissolve the formazan product of MTT. The optical density was measured at 570 nm by a microplate reader (Anthros, Durham, NC, USA). The percentage of cell viability and IC_50_ were calculated according to the manufacturer’s protocol (7sea Biotech). Cell viability = (ODexperiment − ODblank)/(ODcontrol − ODblank) × 100%. Some concentrations of RT were chosen for using in the next experiments.

### 4.6. Nuclear Staining Assay

Hoechst 33342 and PI double staining were used for screening and detecting cell morphology. H460 and BEAS-2B cells were seeded and treated with various concentrations of RT (0, 0.05, 0.1, 1, 5, 10, and 25 µM) for 24 h. Then, cells were stained with 10 µg/mL Hoechst 33342 for 15 min at 37 °C and then stained with 5 µg/mL PI. Fluorescence of nuclear-stained cells was detected randomly using the fluorescent microscope (Olympus IX5, Tokyo, Japan). Nuclear condensation and DNA fragmentation were analyzed as the percentage of apoptotic cells. 

### 4.7. Annexin V-FITC/PI Staining Apoptotic Assay

H460 cells were seeded and treated with various concentrations of RT (0, 0.01, 0.05, 0.1, 1, 5, 10, and 25 µM) for 24 h. After that, cells were collected via centrifugation, washed twice with cold PBS, pH 7.4, and suspended in binding buffer. Then, 5 µL of annexin V-FITC and 1 µL of PI were added to stain the cells for 15 min at room temperature as recommended in the manufacturer’s protocol (ImmunoTools, Friesoythe, Germany). Live, apoptotic, and necrotic cells were analyzed using Guava easyCyte flow cytometer (EMD Millipore, Hayward, CA, USA).

### 4.8. Western Blot Analysis

H460 cells were seeded and treated with various concentrations of RT (0, 1, 5, and 10 µM) for 24 h. The treated cells were collected and lysed with radioimmunoprecipitation assay (RIPA) lysis buffer containing a protease inhibitor cocktail (Roche Diagnostics, Indianapolis, IN, USA) for 1 h on ice. Protein concentrations were determined using the bicinchoninic acid (BCA) assay. All protein samples (70 µg) were run on SDS-polyacrylamide gel electrophoresis and further transferred to 0.45 µm nitrocellulose and 0.2 µm polyvinylidene difluoride (PVDF) membranes (Bio-Rad Laboratories, Hercules, CA, USA). Then, membranes were blocked in Tris-buffer saline containing 0.1% Tween 20 and 5% non-fat dry milk for 2 h at room temperature and incubated with the specific primary antibodies at 4 °C for overnight. The membranes were washed and incubated with horseradish peroxidase (HRP)-conjugated secondary antibody for 2 h at room temperature. Immunoreactive proteins were detected with the enhanced chemiluminescent detection system (Supersignal West Pico, Pierce, Rockford, IL, USA) and subsequently exposed to X-ray film. Protein bands were analyzed using the ImageJ software (version 1.52, National Institutes of Health, Bethesda, MD, USA).

### 4.9. Cycloheximide (CHX) Chasing Assay

H460 cells were seeded and treated with various concentrations of RT (0, 1, 5, and 10 µM) with or without 50 µg/mL CHX for 0, 1, 2, and 4 h. The treated cells were collected and lysed with RIPA lysis buffer containing the protease inhibitor cocktail (Roche Diagnostics, Indianapolis, IN, USA). Western blot analysis was performed for detecting Mcl-1 protein levels. Protein bands were analyzed using the ImageJ software (version 1.52, National Institutes of Health, Bethesda, MD, USA), and the Mcl-1 protein half-life was calculated.

### 4.10. Immunoprecipitation Assay

H460 cells were seeded and treated with 0 and 5 µM of RT for 4 h. The treated cells were collected and lysed with RIPA lysis buffer containing the protease inhibitor cocktail (Roche Diagnostics, Indianapolis, IN, USA). Then, Immunoprecipitation was performed by using Dynabeads™ Protein G Immunoprecipitation Kit from Thermo Fisher Scientific Inc. (Waltham, MA, USA). Magnetic beads were prepared and resuspended with the primary antibody of Mcl-1 in a binding buffer for 10 min. A suspension of the magnetic bead-Ab complex was mixed with lysed protein and incubated at 4 °C overnight to allow Mcl-1 antigen to bind with magnetic bead-Ab complex. After that, the magnetic bead-Ab-Ag complex was washed three times using 200 µL washing Buffer, separated on the magnet between each wash, and the supernatant was removed. Elution Buffer was added for releasing the Ab-Ag complex from magnetic beads. The supernatant contained the Ab-Ag complex was then used to perform Western blot analysis for detecting the ubiquitinated Mcl-1 protein.

### 4.11. Statistical Analysis

The data from three independent experiments (n = 3) was presented as the mean ± standard error of the mean (SEM). Statistical differences between multiple groups were analyzed using an analysis of variance (ANOVA). The p-value of less than 0.05 was considered as statistically significant.

## 5. Conclusions

**Figure 5:**
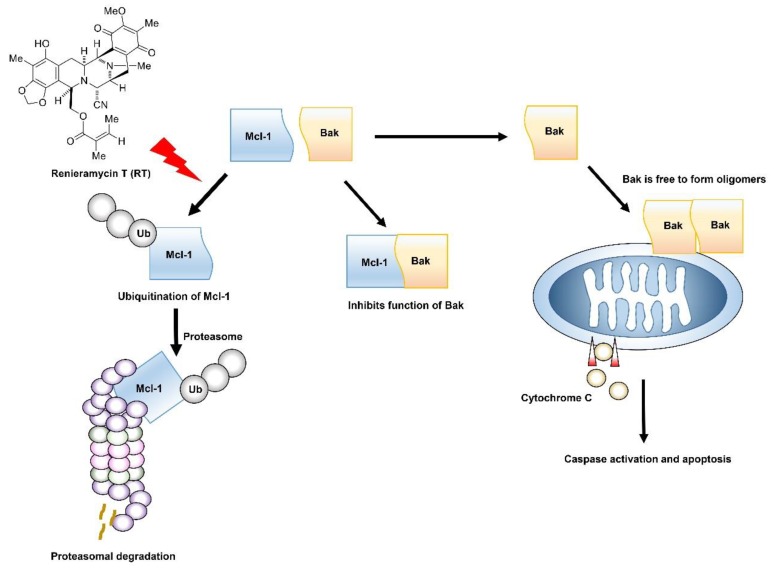
Renieramycin T (RT) enhances apoptosis induction through Mcl-1 proteasomal degradation. Normally, Mcl-1 forms complex with Bak to inhibit its apoptotic function, but when Mcl-1 is degraded through the ubiquitin-mediated proteasomal degradation by treatment of RT, Bak is relieved. Activated Bak forms oligomerization that can permeabilize the outer membrane of mitochondria and release cytochrome c to initiate the apoptosis mechanism.

In conclusion, this study provides supporting evidence for RT to be developed for the anti-cancer therapy in a lung cancer cell model. The RT was shown to be predominantly toxic to lung cancer cells rather than normal epithelial cells in the lung. The mechanism of action of RT is quite specific. The compound mediates Mcl-1 depletion by enhancing the ubiquitin-proteasomal degradation of the protein. As Mcl-1 was demonstrated to control for survival and progression of cancer, these data might be beneficial for highlighting RT as a novel lead compound with supportive information to be further developed for targeted anti-cancer approaches (Figure 5).

## References

[1] Society A.C. (2019). Cancer Facts & Figures 2019.

[2] Chan B.A., Hughes B.G.M. (2015). Targeted therapy for non-small cell lung cancer: Current standards and the promise of the future. Transl. Lung Cancer Res..

[3] Kang M.H., Reynolds C.P. (2009). Bcl-2 inhibitors: Targeting mitochondrial apoptotic pathways in cancer therapy. Clin. Cancer Res..

[4] Thomas S., Quinn B.A., Das S.K., Dash R., Emdad L., Dasgupta S., Wang X.Y., Dent P., Reed J.C., Pellecchia M. (2013). Targeting the Bcl-2 family for cancer therapy. Expert Opin. Ther. Targets.

[5] Del Poeta G., Venditti A., Del Principe M.I., Maurillo L., Buccisano F., Tamburini A., Cox M.C., Franchi A., Bruno A., Mazzone C. (2003). Amount of spontaneous apoptosis detected by Bax/Bcl-2 ratio predicts outcome in acute myeloid leukemia (AML). Blood.

[6] Minn A.J., Rudin C.M., Boise L.H., Thompson C.B. (1995). Expression of bcl-xL can confer a multidrug resistance phenotype. Blood.

[7] Yoshino T., Shiina H., Urakami S., Kikuno N., Yoneda T., Shigeno K., Igawa M. (2006). Bcl-2 expression as a predictive marker of hormone-refractory prostate cancer treated with taxane-based chemotherapy. Clin. Cancer Res..

[8] Chen Y., Gibson S.B. (2017). Mcl-1 is a Gate Keeper Regulating Cell Death in Cancer Cells. J. Clin. Exp. Oncol..

[9] Yang-Yen H.F. (2006). Mcl-1: A highly regulated cell death and survival controller. J. Biomed. Sci..

[10] Chen L., Willis S.N., Wei A., Smith B.J., Fletcher J.I., Hinds M.G., Colman P.M., Day C.L., Adams J.M., Huang D.C. (2005). Differential targeting of prosurvival Bcl-2 proteins by their BH3-only ligands allows complementary apoptotic function. Mol. Cell.

[11] Clohessy J.G., Zhuang J., de Boer J., Gil-Gomez G., Brady H.J. (2006). Mcl-1 interacts with truncated Bid and inhibits its induction of cytochrome c release and its role in receptor-mediated apoptosis. J. Biol. Chem..

[12] Shimazu T., Degenhardt K., Nur E.K.A., Zhang J., Yoshida T., Zhang Y., Mathew R., White E., Inouye M. (2007). NBK/BIK antagonizes MCL-1 and BCL-XL and activates BAK-mediated apoptosis in response to protein synthesis inhibition. Genes Dev..

[13] Willis S.N., Chen L., Dewson G., Wei A., Naik E., Fletcher J.I., Adams J.M., Huang D.C. (2005). Proapoptotic Bak is sequestered by Mcl-1 and Bcl-xL, but not Bcl-2, until displaced by BH3-only proteins. Genes Dev..

[14] Nakano T., Liu D., Nakashima N., Yokomise H., Nii K., Go T., Tarumi S., Matsuura N., Chang S.S., Fujiwara A. (2018). MCL-1 expression of non-small cell lung cancer as a prognostic factor and MCL-1 as a promising target for gene therapy. J. Clin. Oncol..

[15] Quinn B.A., Dash R., Azab B., Sarkar S., Das S.K., Kumar S., Oyesanya R.A., Dasgupta S., Dent P., Grant S. (2011). Targeting Mcl-1 for the therapy of cancer. Expert Opin. Investig. Drugs.

[16] Tanaka N. (2017). The anti-apoptotic protein MCL1, a novel target of lung cancer therapy. J. Cancer Treat. Diagn..

[17] Balakrishnan K., Burger J.A., Wierda W.G., Gandhi V. (2009). AT-101 induces apoptosis in CLL B cells and overcomes stromal cell-mediated Mcl-1 induction and drug resistance. Blood.

[18] Hermanson D.L., Das S.G., Li Y., Xing C. (2013). Overexpression of Mcl-1 confers multidrug resistance, whereas topoisomerase IIbeta downregulation introduces mitoxantrone-specific drug resistance in acute myeloid leukemia. Mol. Pharmacol..

[19] Shuang W., Hou L., Zhu Y., Li Q., Hu W. (2017). Mcl-1 stabilization confers resistance to taxol in human gastric cancer. Oncotarget.

[20] Inuzuka H., Shaik S., Onoyama I., Gao D., Tseng A., Maser R.S., Zhai B., Wan L., Gutierrez A., Lau A.W. (2011). SCF(FBW7) regulates cellular apoptosis by targeting MCL1 for ubiquitylation and destruction. Nature.

[21] Podar K., Gouill S.L., Zhang J., Opferman J.T., Zorn E., Tai Y.T., Hideshima T., Amiot M., Chauhan D., Harousseau J.L. (2008). A pivotal role for Mcl-1 in Bortezomib-induced apoptosis. Oncogene.

[22] Tong J., Wang P., Tan S., Chen D., Nikolovska-Coleska Z., Zou F., Yu J., Zhang L. (2017). Mcl-1 Degradation Is Required for Targeted Therapeutics to Eradicate Colon Cancer Cells. Cancer Res..

[23] Wertz I.E., Kusam S., Lam C., Okamoto T., Sandoval W., Anderson D.J., Helgason E., Ernst J.A., Eby M., Liu J. (2011). Sensitivity to antitubulin chemotherapeutics is regulated by MCL1 and FBW7. Nature.

[24] Ruiz-Torres V., Encinar J.A., Herranz-Lopez M., Perez-Sanchez A., Galiano V., Barrajon-Catalan E., Micol V. (2017). An Updated Review on Marine Anticancer Compounds: The Use of Virtual Screening for the Discovery of Small-Molecule Cancer Drugs. Molecules.

[25] Jimenez P.C., Wilke D.V., Costa-Lotufo L.V. (2018). Marine drugs for cancer: Surfacing biotechnological innovations from the oceans. Clinics.

[26] Chamni S., Sirimangkalakitti N., Chanvorachote P., Saito N., Suwanborirux K. (2017). Chemistry of Renieramycins. 17. A New Generation of Renieramycins: Hydroquinone 5-O-Monoester Analogues of Renieramycin M as Potential Cytotoxic Agents against Non-Small-Cell Lung Cancer Cells. J. Nat. Prod..

[27] Hamann M.T. (2003). Enhancing marine natural product structural diversity and bioactivity through semisynthesis and biocatalysis. Curr. Pharm. Des..

[28] Scott J.D., Williams R.M. (2002). Chemistry and biology of the tetrahydroisoquinoline antitumor antibiotics. Chem. Rev..

[29] Frincke J.M., Faulkner D.J. (1982). Antimicrobial metabolites of the sponge *Reniera* sp.. J. Am. Chem. Soc..

[30] He H.Y., Faulkner D.J., Shumsky J.S., Hong K., Clardy J. (1989). A sesquiterpene thiocyanate and three sesquiterpene isothiocyanates from the sponge *Trachyopsis aplysinoides*. J. Org. Chem..

[31] Amnuoypol S., Suwanborirux K., Pummangura S., Kubo A., Tanaka C., Saito N. (2004). Chemistry of renieramycins. Part 5. Structure elucidation of renieramycin-type derivatives O, Q, R, and S from thai marine sponge *Xestospongia* species pretreated with potassium cyanide. J. Nat. Prod..

[32] Davidson B.S., Renieramycin G. (1992). A new alkaloid from the sponge *Xestospongia caycedoi*. Tetrahedron Lett..

[33] Saito N., Hiramatsu A., Hirade H., Kubota M., Toyoshima R., Fujino A., Sirimangkalakitti N., Suwanborirux K., Suwanborirux G.P. (2017). Chemistry of Renieramycins. 16. Structure of 7-Desmethylrenieramycin O (= 14α-Hydroxyrenieramycin S) from Blue Sponge, *Xestospongia* sp.. Heterocycles.

[34] Suwanborirux K., Amnuoypol S., Plubrukarn A., Pummangura S., Kubo A., Tanaka C., Saito N. (2003). Chemistry of renieramycins. Part 3. isolation and structure of stabilized renieramycin type derivatives possessing antitumor activity from Thai sponge *Xestospongia* species, pretreated with potassium cyanide. J. Nat. Prod..

[35] Tatsukawa M., Punzalan L.L.C., Magpantay H.D.S., Villaseñor I.M., Concepcion G.P., Suwanborirux K., Yokoya M., Saito N. (2012). Chemistry of renieramycins. Part 13: Isolation and structure of stabilized renieramycin type derivatives, renieramycins W–Y, from Philippine blue sponge *Xestospongia* sp., pretreated with potassium cyanide. Tetrahedron.

[36] Parameswaran P.S., Naik C.G., Kamat S.Y., Pramanik B.N. (1998). Renieramycins H and I, two novel alkaloids from the sponge *Haliclona cribricutis* Dendy. Indian J. Chem. Sect. B.

[37] Pettit G.R., Knight J.C., Collins J.C., Herald D.L., Pettit R.K., Boyd M.R., Young V.G. (2000). Antineoplastic Agents 430. Isolation and Structure of Cribrostatins 3, 4, and 5 from the Republic of Maldives *Cribrochalina* Species. J. Nat. Prod..

[38] Oku N., Matsunaga S., van Soest R., Fusetani N., Renieramycin J. (2003). A highly cytotoxic tetrahydroisoquinoline alkaloid, from a marine sponge *Neopetrosia* sp.. J. Nat. Prod..

[39] Daikuhara N., Tada Y., Yamaki S., Charupant K., Amnuoypol S., Suwanborirux K., Saito N. (2009). Chemistry of renieramycins. Part 7: Renieramycins T and U, novel renieramycin–ecteinascidin hybrid marine natural products from Thai sponge *Xestospongia* sp.. Tetrahedron Lett..

[40] He W., Zhang Z., Ma D. (2019). A Scalable Total Synthesis of the Antitumor Agents Et-743 and Lurbinectedin. Angew. Chem. Int. Ed..

[41] Akgul C. (2009). Mcl-1 is a potential therapeutic target in multiple types of cancer. Cell. Mol. Life Sci..

[42] Abid M., Sonawane Y.A., Contreras J.I., Rana S., Natarajan A. (2017). Recent Advances in Cancer Drug Development: Targeting Induced Myeloid Cell Leukemia-1 (Mcl-1) Differentiation Protein. Curr. Med. Chem..

[43] Tron A.E., Belmonte M.A., Adam A., Aquila B.M., Boise L.H., Chiarparin E., Cidado J., Embrey K.J., Gangl E., Gibbons F.D. (2018). Discovery of Mcl-1-specific inhibitor AZD5991 and preclinical activity in multiple myeloma and acute myeloid leukemia. Nat. Commun..

[44] Kao S.-H., Wang W.-L., Chen C.-Y., Chang Y.-L., Wu Y.-Y., Wang Y.-T., Wang S.-P., Nesvizhskii A.I., Chen Y.-J., Hong T.-M. (2015). Analysis of Protein Stability by the Cycloheximide Chase Assay. Bio-Protocol.

[45] Mojsa B., Lassot I., Desagher S. (2014). Mcl-1 ubiquitination: Unique regulation of an essential survival protein. Cells.

[46] Green D.R., Kroemer G. (2009). Cytoplasmic functions of the tumour suppressor p53. Nature.

[47] Senturk E., Manfredi J.J. (2013). p53 and cell cycle effects after DNA damage. Methods Mol. Biol..

[48] Hemann M.T., Lowe S.W. (2006). The p53-Bcl-2 connection. Cell Death Differ..

[49] Shamas-Din A., Kale J., Leber B., Andrews D.W. (2013). Mechanisms of action of Bcl-2 family proteins. Cold Spring Harb. Perspect. Biol..

[50] Zhang H., Guttikonda S., Roberts L., Uziel T., Semizarov D., Elmore S.W., Leverson J.D., Lam L.T. (2011). Mcl-1 is critical for survival in a subgroup of non-small-cell lung cancer cell lines. Oncogene.

[51] Chowdry R., Sica G.L., Kim S., Chen Z., Goodman A., Alexis D., Deng X., Owonikoko T.K. (2016). Phosphorylated Bcl-2 and Mcl-1 as prognostic markers in small cell lung cancer. Oncotarget.

[52] Saito N., Tanaka C., Koizumi Y.-I., Suwanborirux K., Amnuoypol S., Pummangura S., Kubo A. (2004). Chemistry of renieramycins. Part 6: Transformation of renieramycin M into jorumycin and renieramycin J including oxidative degradation products, mimosamycin, renierone, and renierol acetate. Tetrahedron.

[53] Jia J., Chen R., Liu H., Li X., Jia Y., Chen X. (2016). Asymmetric synthesis of (−)-renieramycin T. Org. Biomol. Chem..

[54] Kimura S., Saito N. (2018). Construction of the Pentacyclic Core and Formal Total Synthesis of (rac)-Renieramycin T. ChemistryOpen.

[55] Yokoya M., Toyoshima R., Suzuki T., Le V.H., Williams R.M., Saito N. (2016). Stereoselective Total Synthesis of (−)-Renieramycin, T. J. Org. Chem..

[56] Chantarawong W., Chamni S., Suwanborirux K., Saito N., Chanvorachote P. (2019). 5-O-Acetyl-Renieramycin T from Blue Sponge *Xestospongia* sp. Induces Lung Cancer Stem Cell Apoptosis. Mar. Drugs.

[57] Bipasha B., Sudheer Shenoy P. (2014). Stem Cell versus Cancer and Cancer Stem Cell: Intricate Balance Decides Their Respective Usefulness or Harmfulness in the Biological System. J. Stem Cell Res. Ther..

[58] Jordan C.T., Guzman M.L., Noble M. (2006). Cancer stem cells. N. Engl. J. Med..

[59] Reya T., Morrison S.J., Clarke M.F., Weissman I.L. (2001). Stem cells, cancer, and cancer stem cells. Nature.

